# The implementation of trauma resuscitation procedures within a field hospital setting in Turkey: an examination of a distinctive collaborative approach

**DOI:** 10.1186/s13049-023-01116-7

**Published:** 2023-09-28

**Authors:** Nihat Mujdat Hokenek, Alican Barış, Mehmet Fatih İnecikli

**Affiliations:** 1 Department of Emergency Medicine, University of Health Sciences, Kartal Dr. Lutfi Kirdar City Hospital, Istanbul, Turkey, Kartal, Turkey; 2 Department of Orthopaedics and Traumatology, University of Health Sciences, İstanbul Physical Therapy And Rehabilitation Training And Research Hospital, İstanbul, Turkey; 3 Department of Radiology, Bursa Uludag University, School of Medicine, Bursa, Turkey

On February 6, 2023, Pazarcık (Kahramanmaraş) and Elbistan (Kahramanmaraş) in Turkey experienced two seismic events, characterized by moment magnitudes (Mw) of 7.7 and 7.6, respectively [1]. Based on authoritative data, the seismic event resulted in a casualty count exceeding 50,000 individuals. Upon the immediate occurrence of the disaster, healthcare professionals nationwide promptly offered their voluntary services to journey to the impacted region.

On the subsequent day following the occurrence of the calamity, a field hospital was promptly established within the premises of the extensively impaired “Hatay Training and Research Hospital” located in Hatay. The individuals who were saved from the debris were transferred via ambulance from the area affected by the catastrophe to the designated medical facility. This correspondence aims to delineate the initial medical procedures conducted on patients who were extricated from the debris at the field hospital.

**Fig. 1:**
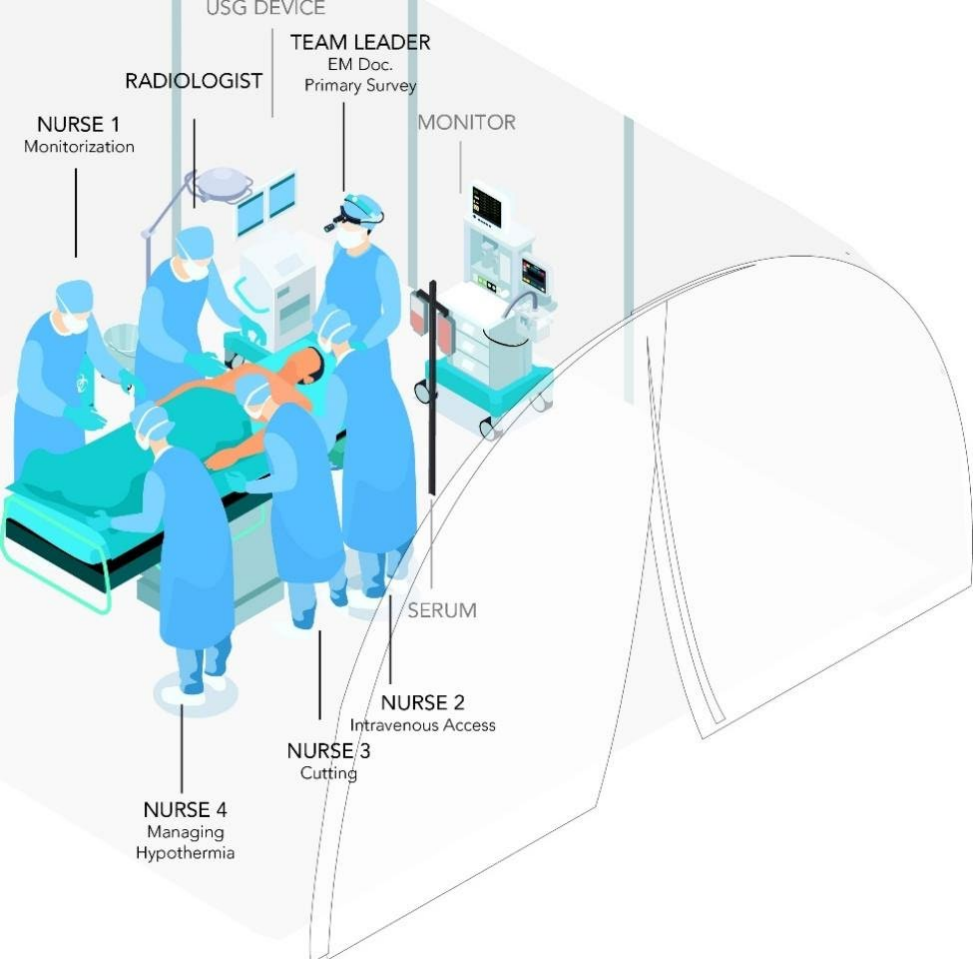
Organization of the trauma team in the field hospital

The organizational structure and task allocation for the red zone of the field hospital were devised by an emergency medicine specialist (EMS). As a result, trauma teams were established. These teams consisted of four registered nurses, one emergency medicine specialist, and one radiologist. As depicted in Fig. 1, nurses were tasked with the responsibilities of cutting, monitorizing patient vital signs, ensuring proper temperature regulation, and establishing intravenous access. During the initial assessment conducted by the Emergency Medical Services (EMS), the radiologist conducted an e-fast examination and Doppler ultrasonography (USG) on the extremities affected by cold exposure. Following that, the radiologist employed ultrasonography to perform the insertion of a central venous catheter. During the initial assessment, EMS communicated the examination results to the secretary, who duly documented them. The individuals who underwent resuscitation and achieved hemodynamic stability were transported via helicopter to a tertiary care facility located in a different city [2].

Due to our methodology, prompt monitoring, rapid warming, and expedited access to intravascular regions were achieved for patients affected by the earthquake. Furthermore, the expeditious nature of the ultrasonographic examination facilitated prompt diagnosis, thereby enabling efficient patient transfers to appropriate medical facilities. Our findings indicate that our methodology can be applicable for conducting primary assessments of patients affected by disasters.

### Electronic supplementary material

Below is the link to the electronic supplementary material.

Supplementary Material 1
