# Supplementary material for: The implementation of trauma resuscitation procedures within a field hospital setting in Turkey: an examination of a distinctive collaborative approach
Source: Scand J Trauma Resusc Emerg Med. 2023 Sep 28;31:51. doi: 10.1186/s13049-023-01116-7 (PMC10540320; doi:10.1186/s13049-023-01116-7)
Supplement: Supplementary file 1 — Supplementary Material 1 [file 13049_2023_1116_MOESM1_ESM.docx]

03/09/2023

Dear Editor in Chief ;

We would like to present a letter to the editor named *“ Trauma resuscitation in a field hospital in Turkey: What we learned from the earthqu ake?’’* for your consideration.

As you know, there was a great earthquake disaster in our region. In the disaster area, a serious chaos environment was waiting for the volunteer health workers who went there. Although we were trained for such situations, the realities of the field were different than they should have been. In this letter, we have expressed in our own experience how the primary care of the trauma patient can be done in this sense, under the conditions at hand. We hope that our approach will contribute to the current literature as an experience. Evaluation of this letter in your journal is very important for our healthcare professionals working in the region in terms of entering their work into the literature. We offer our respects in the hope that a pioneering journal like yours will evaluate this bitter experience.

Please find attached the paper by Nihat Müjdat Hökenek, M.D.; Alican Barış, M.D.; Mehmet Fatih İnecikli, M.D. which we would like to submit for possible publication to the ‘’*Scandinavian Journal of Trauma, Resuscitation and Emergency Medicine’’*.

We confirm that this work is original and has not been published elsewhere nor is it currently under consideration for publication elsewhere.

We give our permission to the Publisher to use any published material.

The authors have no commercial associations or sources of support that might pose a conflict of interest.

For any information concerning this manuscript, please contact me preferably by at e-mail [nihathokenek@gmail.com](mailto:nihathokenek@gmail.com) .

Thank you for your consideration of this manuscript.
I look forward to hearing from you soon.

Yours sincerely,

Corresponding Author:

Associate Prof. Dr. Nihat Müjdat Hökenek

Advanced Trauma Life Support Instructor

Emergency Medicine Specialist

Kozyatağı mah. Bayar cad. 34/9 Kadıköy/İstanbul

E-mail: nihathokenek@gmail.com

GSM:+905553201419

University of Health Sciences, Kartal Dr. Lütfi Kırdar City Hospital

Department of Emergency Medicine
